# To Lead and To Lag – Forward and Backward Recalibration of Perceived Visuo-Motor Simultaneity

**DOI:** 10.3389/fpsyg.2012.00599

**Published:** 2013-01-22

**Authors:** Marieke Rohde, Marc O. Ernst

**Affiliations:** ^1^Department of Cognitive Neurosciences, University of BielefeldBielefeld, Germany; ^2^Cognitive Interaction Technology Centre of Excellence, University of BielefeldBielefeld, Germany

**Keywords:** time perception, visuo-motor integration, temporal recalibration, multisensory perception, simultaneity perception

## Abstract

Studies on human recalibration of perceived visuo-motor simultaneity so far have been limited to the study of recalibration to movement-lead temporal discrepancies (visual lags). We studied adaptation to both vision-lead and movement-lead discrepancies, to test for differences between these conditions, as a leading visual stimulus violates the underlying cause-effect structure. To this end, we manipulated the temporal relationship between a motor action (button press) and a visual event (flashed disk) in a training phase. Participants were tested in a temporal order judgment task and perceived simultaneity (PSS) was compared before and after recalibration. A PHANToM©force-feedback device that tracks the finger position in real time was used to display a virtual button. We predicted the timing of full compression of the button from early movement onset in order to time visual stimuli even before the movement event of the full button press. The results show that recalibration of perceived visuo-motor simultaneity is evident in both directions and does not differ in magnitude between the conditions. The strength of recalibration decreases with perceptual accuracy, suggesting the possibility that some participants recalibrate less because they detect the discrepancy. We conclude that the mechanisms of temporal recalibration work in both directions and that there is no evidence that they are asymmetrical around the point of actual simultaneity, despite the underlying asymmetry in the cause-effect relation.

## Introduction

When determining the timing of multisensory events, our brains have to compensate for cross-sensory latencies that stem from physical sources (e.g., light travels faster than sound) as well as physiological sources (e.g., differences in sensory transduction or neural transmission times). A growing body of evidence shows that the mechanisms of latency compensation are plastic and that they can be recalibrated by exposing participants for some period of time to a systematic small temporal discrepancy between uni-modal events. Temporal recalibration of this kind has been shown, for instance, for the perception of audio-visual, audio-tactile, and visuo-tactile simultaneity (e.g., Fujisaki et al., 1994; Keetels and Vroomen, 2008; Di Luca et al., 2009).

The perceived order of a voluntary movement event and an external sensory event seems to be no exception from this rule. Stetson et al. (2006) have shown that humans recalibrate to partially compensate for a 100 ms lag between a button press and a visual flash. Similar results were reported in experiments with rhythmic finger tapping, including studies of sensory-motor recalibration in other modality pairs (tactile-motor, auditory-motor) and where transfer across modalities was observed (Heron et al., 2009; Sugano et al., 2010; Keetels and Vroomen, 2012; Sugano and Vroomen, 2012). Heron et al. (2009) could show that visuo-motor temporal recalibration weakens with increasing temporal discrepancy. Arnold et al. (2012) have shown that this constraint of temporal proximity is relative to the time of button press, not to the time of movement planning or movement onset. Yet, these kinds of studies have so far been limited to scenarios where the movement event leads the temporal order^1^. It is not clear, however, whether adaptation where an external sensory event precedes a voluntary movement is possible and, if it is, whether it follows the same rules as adaptation to movement-lead discrepancies. This is an interesting question because of the causal relationship that usually is accompanied with such sensory-motor events, i.e., a voluntary button press may trigger a flash but not vice versa. Given this rationale, a possible hypothesis is that it is not possible or more difficult to adapt if a flash precedes the movement event because of a violation of the naturally occurring causal relationship. By contrast, given that mechanisms of sensory-motor recalibration tend to operate symmetrically in space, a different hypothesis would be that recalibration should work symmetrically in time as well. Here we designed an experiment to empirically test these two alternative hypotheses.

Evidence in the literature that supports the asymmetry hypothesis stems from several sources. For instance, differences in processing around the point of actual simultaneity have been found in audio-visual speech perception, where subjects tolerate much larger auditory lags than visual lags, leading to an asymmetric temporal window of integration (van Wassenhove et al., 2007). Even though there are also functional explanations for this asymmetry, the authors think it is possible that this asymmetry could arise simply from differences in uni-modal neural processing. Such asymmetries could in principle be found in any modality pair. Also, the above-mentioned possible causal relation between a voluntary movement event and a subsequent sensory event could lead to asymmetry around the actual point of simultaneity. Haggard et al. (2002) have shown that, if a sensory event systematically follows shortly after a voluntary movement, it will be interpreted as sensory feedback (intentional binding) and both will be perceived closer together in time then when movement is not voluntary and intentional (Haggard et al., 2002, cf. also Eagleman and Holcombe, 2002). This intentional binding appears to be a special case of causal binding that occurs whenever humans assume a causal link between two events (Buehner and Humphreys, 2009). Intentional or causal binding thus appears to strengthen the “unity assumption” (Welch and Warren, 1980), i.e., the assumption that events belong together and originate from a common source, which is a key requirement for multisensory integration. If a sensory event precedes the voluntary movement event, however, the underlying cause-effect relationship for intentional binding is violated, which may decrease the unity assumption and slow down or even prevent multisensory recalibration. Thirdly, during growth and development, compensation for longer visual feedback latencies (longer nerve conductance times) may be required, whereas a shortening of neural conductance latencies is, ecologically speaking, not to be expected.

There are, however, also arguments in favour of the symmetry hypothesis. For instance, a shortening of visuo-motor latencies, even if physiologically implausible, can occur in our interaction with digital technology, where the reaction, e.g., the appearance of a letter on the screen, may be delayed with respect to the button press on the keyboard. If we are able to adapt to this kind of delay, we should also be accustomed to a re-adaptation in the reverse direction when stopping interaction with the device, even though there could be absolute limits on this reverse adaptation. Also, simplicity favors delay compensation mechanisms that are general and thus symmetrical, such as the Kalman Filter model suggested by (Burge et al., 2008) or the Smith predictor model of cerebellar visuo-motor control in motor behavior (e.g., Miall et al., 1993), where sensory-motor latency compensation is implemented separately from a plasticity rule that estimates sensory-motor delays to be compensated from experience.

Researchers attempting to empirically settle this question by also studying adaptation to vision-lead temporal discrepancies between voluntary movement and vision will face a technical difficulty. In order to time the presentation of a visual stimulus before a voluntary movement event, the experimenter has to know or to predict when a subject will perform the action. This problem has been elegantly solved by Stetson et al. (2006). The authors kept a running average of participants’ reaction times to an external cue event. They were thus able to present visual stimuli from a range of temporal discrepancies that was symmetrical around the point of actual simultaneity of button press and visual event, using the average reaction time in previous trials as a predictor for the timing of the next button press. Recalibration also occurs in the absence of an external cue event, as a second experiment by Stetson et al. (2006) confirmed. In this experiment, participants themselves chose the timing of repeated button presses. The time of a future button press was then predicted from the relative timing of previous button presses. Similarly, Arnold et al. (2012) used a leading button release to time visual stimuli to occur before a second button press at the end of a ballistic reach. These kinds of prediction, however, are likely not accurate enough on a trial-by-trial basis to time a temporal discrepant recalibration stimulus.

For the current study, we developed a new method to test whether participants recalibrate equally to the presence of vision-lead and movement-lead temporal discrepancies. We used a haptic device (PHANToM©force-feedback device, Sensable Inc.) to display a virtual button and tracked participants’ finger movement online. Using an adaptive threshold method (cf. Materials and Methods), we predicted the moment of full compression of the virtual button in real time. We were able to predict the button press quite precisely within about 100 ms such that we could reliably present visual-motor stimuli with vision leading by 100 ms with respect to the movement event (the full button press). We could thus compare adaptation to vision-lead and movement-lead temporal discrepancies within a window of ±100 ms.

## Materials and Methods

### Experimental setup

**Figure 1:**
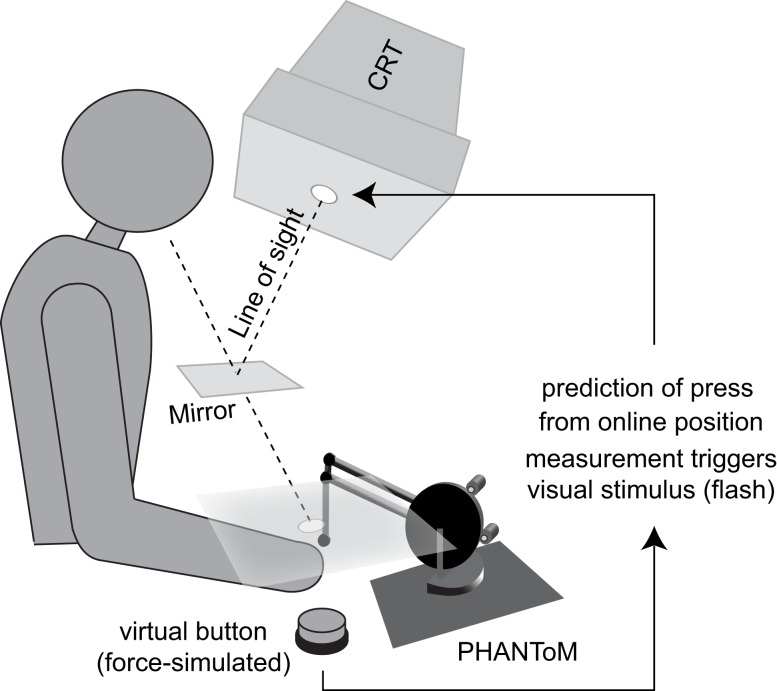
**Schematic of the experimental setup**.

**Figure 2:**
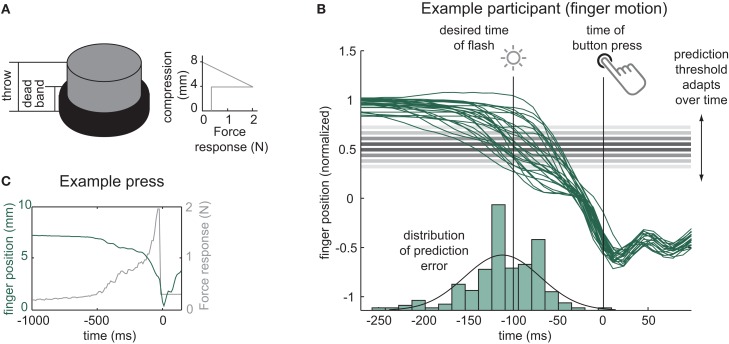
**The virtual button and prediction method**. **(A)** Force-response of the virtual button. **(B)** Example finger movement trajectories in the vertical dimension (green solid lines; 1 unit~4 mm, normalized for initial finger height, cf. main text) and prediction error over 333 trials (green histogram at the bottom) for a l = −100 ms (vision-lead) stimulus in an example participant. The adaptive threshold moves up and down according to the sign of the error on a previous trial. **(C)** Example button press (green) and force-response (grey) across time.

Participants were seated in a dark room and placed their head in a chin-rest, looking down into the direction of their hands. The hands were occluded from vision by a mirror (see Figure 1). Participants’ right lower arm rested on a board and the right index finger was attached to a PHANToM force-feedback device. The device simulated a virtual button (mass *m* = 0.1 kg) with a throw of 8 mm, which contained a 4 mm spring (spring constant *k* = 500 kg/s^2^) plus a dead-band of 4 mm (see Figure 2A). A small restoring force (0.3 N) pressed the button back up after full compression (see Figures 2A,C). Participants rested with their finger on the button and did not receive visual feedback about the position or compression of the button. Additionally, a haptically displayed virtual object directly above the button blocked participants from raising the right index finger higher than the height of the decompressed button.

The vertical displacement of the participant’s finger during the button press was tracked in real time, in order to predict the timing of the full compression of the button from early movement onset (cf. following section). The initial resting height on the button varied slightly from trial to trial as participants started a trial resting with the finger on the button. The top part of the button is compliant and thus small differences in the resting force applied by subjects will lead to slight differences in the resting position. Especially when predicting large negative lags, this variability can lead to early alarms if a participant already compresses the button a bit when resting at the beginning of the trial. The tracked vertical position was therefore normalized for the prediction algorithm to the distance between the initial resting position and the entering of the dead-band (cf. Figure 2B, green trajectories), which comprises approximately the 4 mm length of the spring.

**Figure 3:**
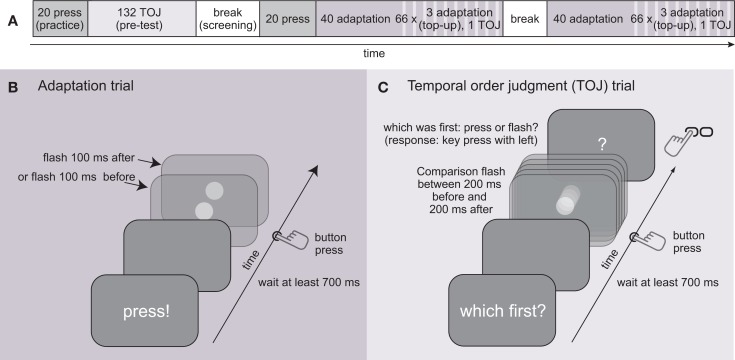
**Procedure and task**. **(A)** Timeline of one experimental session. **(B)** In adaptation trials, participants are presented with either a vision-lead or movement-lead 100 ms discrepancy. **(C)** In temporal order judgment (TOJ) trials, participants respond with a key press of their left hand which one they perceived to occur first – button press or flashed disk.

Visual probe stimuli were projected into participants’ field of view using a CRT monitor mounted upside-down above the mirror. The visual flash stimuli were white disks of 1.5° visual angle on a 50% gray background. At no time during the experiment did the participants receive any visual information beside this flash and instructions printed on the screen (cf. Procedure and Figure 3). The flash was projected into the area where participants pressed the button but was not spatially aligned with the finger. The refresh rate of the monitor was 90 Hz and stimuli were flashed for one frame (i.e., ∼11 ms) upon button press.

The setup has an inherent endpoint-to-endpoint delay of 34.5 ± 7 ms interquartile range (IQR) between a button press in the real world and the display of a visual flash on the screen triggered by the button press. This was measured using two photodiodes and a method similar to that described in Di Luca (2010). In the notation used in this paper the system latency is not yet subtracted when computing the stimulus onset asynchronies (SOAs), i.e., a baseline visuo-motor lag l = 0 corresponds to a scenario where a button triggers a visual stimulus that then flashes on the screen 34.5 ms later (visual lag l is defined as l = *t*_v_ − *t*_m_ where t_m_ is the time of full decompression of the button and *t*_v_ the time of the visual flash).

### Prediction method

The vertical position of the right index finger was tracked with a frequency of 90 Hz to predict the moment of full compression of the button (cf. Figure 2A).

Besides being precise, there are a number of requirements the prediction method has to fulfill. It has to be simple, in order to compute in real time; it should be robust because motion profiles for the button presses vary both within and between participants; and it should be unbiased, i.e., it should be more or less equally prone to predicting too early or too late. We found that, by and large, an adaptive threshold method performed well according to all of these criteria. An array of thresholds corresponding to the different SOAs (cf. Procedure) was initialized using the median position of the finger during the 20 practice button presses as starting threshold. Afterward, this threshold moved up or down with a step size of 0.05 units (∼0.2 mm). The direction of the step depended on the sign of the error of the previous prediction, i.e., it moved up if prediction had been too late and it moved down if prediction had been too early. The mean IQR across participants and conditions with which this method could predict the target recalibration discrepancy of l = −100 ms was 61 ms (range of IQR of prediction error: 22–122 ms).

### Procedure and task

Ten paid volunteers (7 female; Average age 25.7, age range 21–38; all right-handed as by self-report) were tested in two conditions on different days: movement-lead adaptation (l = 100 ms) and vision-lead adaptation (l = −100 ms). The order of these conditions was counter-balanced across participants. The experiments were approved by the Ethics Committee of the University Clinics Tübingen, Germany. All participants signed informed consent forms and were naïve to the purpose of the experiment. Each session lasted for 60–90 min and consisted of one pre-test block (block 1) and two adaptation/post-test blocks (blocks 2 and 3; cf. Figure 3A).

Participants were instructed to wait for at least 700 ms and as long as they wanted after a trial started before pressing the button. This minimum waiting period was introduced for two reasons. Firstly, the predictor had to be given time to generate its prediction. Secondly, we wanted to avoid that button presses are simple reactions to an external trigger (initiation of trial). Self-initiation of an action alters both temporal processing (e.g., Jenkins et al., 2000) and behavior (e.g., Welchman et al., 2010). If the button was pressed too early, the words “too early” were projected into participants’ field of view and the trial was repeated. Blocks 1 and 2 started with some training of 20 button presses that triggered a task-unrelated auditory signal, to initiate the predictor and habituate participants to the required minimum waiting time of 700 ms.

In Block 1, participants were exposed to temporal order judgment (TOJ) trials only (cf. Figure 3C). A question mark was displayed and participants had to make a forced choice decision about the temporal order (TOJ) whether they had perceived the visual stimulus to have occurred before or after the button press. They were instructed to judge the timing of the visual stimulus compared to the time when they fully compressed it, after entering the dead-band that was haptically clearly perceptible (cf. Figure 2C). They gave their response by pressing a response key with their left hand. Participants were tested for 12 repetitions in the TOJ task with visual comparison stimuli aimed at the following visual lags l: [−200, −150, −100, −67, −33, 0, 33, 67, 100, 150, 200] ms. Values from the negative range were predicted from early movement onset (cf. previous section). As mentioned above, the prediction naturally is not always perfect but may contain some prediction error. If, due to these errors, the effective SOA was closer to a different value from the range of target SOAs, planned comparisons for future trials were rearranged online to ensure an overall balanced presentation of SOAs. A psychometric function in form of a cumulative Gaussian was fit to the responses to the TOJ task using the Matlab toolbox *psignifit* (Wichmann and Hill, 2001a,b) to derive the point of subjective simultaneity (PSS) and the just noticeable difference (JND). PSS and JND were the only free parameters. Participants with a JND > 150 ms in their first block were discarded from the experiment, as the narrow range of SOAs around the PSE does not allow for a reliable estimation of the entire psychometric curves for participants with lower perceptual precision.

In Blocks 2 and 3, participants were first exposed to 40 adaptation trials (Figure 3B) with the respective lags l = −100 (vision-lead) or l = 100 (movement-lead), after which they were again tested with the TOJ task (six repetitions per blocks 2 and 3; cf. Figure 3A), exposing them to three top-up adaptation trials in between each TOJ trial. The noise on the temporal discrepancy in the vision-lead adaptation condition, due to prediction errors, was mirrored across the l = 0 point for the movement-lead condition to assure comparability of the two conditions. If the predictor failed to predict a button press before it occurred (15% of training trials), no visual stimulus was displayed in adaptation trials.

## Results

The PSS in the pre-test of the first session was not significantly different from the zero lag SOA at l = 0. There was a small non-significant bias of −11 ± 12 ms (SEM) toward the vision-lead discrepancy [*t*-test: *p* = 0.378, *t*(9) = 0.9], which may reflect the fact that small system latencies are not corrected for (cf. see Materials and Methods). JNDs were on average 70 ± 4 ms (SEM) across all subjects and conditions and there were no significant differences between conditions.

**Figure 4:**
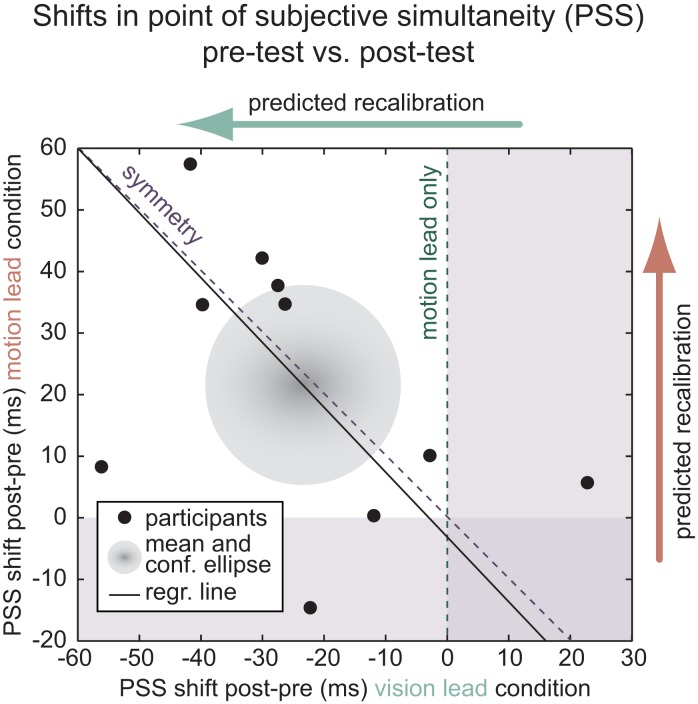
**Recalibration results**. Individual participants (black dots); group mean and confidence ellipse and regression line (black). Recalibration was observed in both the vision-lead and movement-lead conditions (confidence ellipse in white quadrant) and it was not biased to either side of the inverse diagonal representing symmetry of recalibration mechanisms.

Recalibration was computed by subtracting the pre-test PSS from the post-test PSS. A significant recalibration effect could be found in both the vision-lead condition [recalibration: −24 ± 7 ms (SEM), *p* = 0.008, *t*(9) = 3.4] and in the movement-lead condition [recalibration: 22 ± 7 ms (SEM), *p* = 0.015, *t*(9) = 3.0]. Figure 4 depicts the recalibration observed for individual participants as well as the group mean, the confidence ellipse, and a regression line. Paired sample *t*-tests confirmed that there was a significant difference between the two conditions within participants [*p* = 0.004, *t*(9) = 3.8] and that, inverting the sign of recalibration in the vision-lead condition, the magnitude of recalibration did not differ between the two conditions [*p* = 0.806, *t*(9) = 0.3]. This last result supports the hypothesis that recalibration may indeed be symmetrical.

To further assess the symmetry of recalibration in the two conditions, we performed a Deming regression (total least squares fit) of the recalibration in the movement-lead condition to recalibration in the vision-lead condition (see Figure 4). This yielded an intercept *a* = −3 ms (confidence interval: −37, 32 ms) and a slope *b* = −1.05 (confidence interval: −2.22, 0.11 ms). The fitted slope is very close to −1 (perfect symmetry). However, given the limited number of participants and the variability of recalibration effect size, the possibility of asymmetry, i.e., that there is stronger recalibration in the case of movement-lead adaptation, cannot be ruled out.

Taken together, these analyses show that there is recalibration in both the vision-lead and the movement-lead condition. They provide no evidence against the symmetry hypothesis.

**Figure 5:**
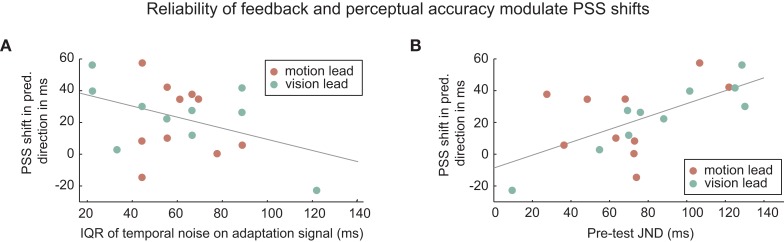
**Reliability of feedback and perceptual accuracy modulate PSS shifts**. PSS-shifts in the predicted direction (i.e., negative for vision-lead) as a function of (A) temporal accuracy of recalibration signal and (B) perceptual precision (pre-test JND). Grey solid lines are least square regression lines.

The size of the recalibration effect we observed was lower than in previous studies, where participants exposed to a 100 ms movement-lead delay recalibrated their PSS between ca. 30 ms (Heron et al., 2009; Sugano et al., 2010) and 44 ms (Stetson et al., 2006). A possible reason for this could be differences in the reliability of the error feedback (i.e., the temporal discrepancy). The prediction method used in our paradigm introduces temporal noise that previous studies on adaptation to movement-lead discrepancies did not have. Burge et al. (2008) found that the rate of adaptation in visuo-motor control decreases with the amount of noise in the error feedback (i.e., the feedback about the temporal discrepancy as measured by the JND) in order to obtain statistically optimal learning of a new sensory-motor mapping. Furthermore, they showed that in a stable world in which the mapping is more predictable the learning rate is reduced. This kind of approach would predict an anti-correlation between the size of the recalibration effect and both the temporal spread of the adaptation signal and the JND. However, there is no significant anti-rank-correlation of recalibration and temporal accuracy of the adaptation signal (*p* = 0.302), even if there is a possible trend in the predicted direction (Figure 5, left). Concerning perceptual precision, rather than the predicted anti-correlation, there is a significant rank-correlation of recalibration and JND (Spearman’s ρ = 0.54, *p* = 0.014), suggesting that precision of one’s own estimate impacts negatively on the amount or speed of recalibration (Figure 5, right). This suggests a different explanation for the lower recalibration effect size. It is possible that participants with a low JND were better able to detect the temporal discrepancy between the movement event and the visual event, which may have decreased the unity assumption. In this case, the screening for especially precise participants (JND < 150 ms) could explain this weaker recalibration.

**Figure 6:**
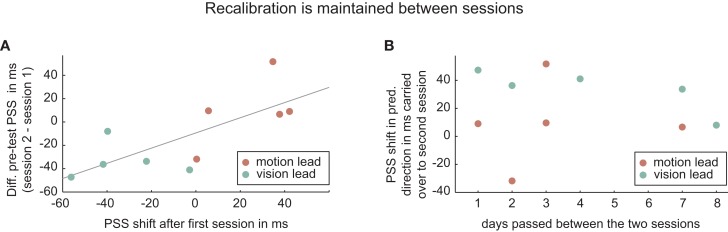
**PSS-shifts are maintained across sessions (A)**. This carry-over of recalibration does not appear to decay over time **(B)**. Grey solid line is least square regression line.

Another observation that was not expected was that the recalibration effect in the first session was nearly fully carried over into the pre-test of the second session. Figure 6 (left) depicts the significant correlation (Pearson’s *r* = 0.73; *p* = 0.016) between PSS shift as an effect of training in the first session and the difference between the pre-tests in both sessions. This carry-over effect does not appear to be related to the amount of time that had passed between the sessions (Figure 6, right, rank-correlation *p* = 0.528). The preservation of recalibration across sessions was an unexpected result. We had assumed that, interacting in real time with the real world for at least 24 h, the new temporal relationship learned in the training would be quickly unlearned. However, these data suggest that the learning is highly context-specific (we used the same setup, experimental room, stimuli, etc. in both sessions) and remains present in our setup despite having had plenty of experience for hours and days with sensory-motor stimuli without temporal delay in the natural world.

A possible concern with this carry-over effect is that adaptation to vision-lead occurs only or predominantly if vision-lead adaptation is performed in the second session, simply as a return to the original state, i.e., that there is no real bi-directionality of the recalibration mechanisms. As Figure 6A illustrates, this is not the case. The recalibration in the first session is in all cases in the predicted direction (green dots: left of vertical zero-line; red dots: right of vertical zero-line). Indeed, even analyzing recalibration just in the first session (with five subjects), recalibration in the vision-lead condition is already significant [recalibration vision-lead: −33 ± 8 ms (SEM), *p* = 0.024, *t*(4) = 3.5; recalibration movement-lead: 24 ± 8 ms (SEM), *p* = 0.024, *t*(4) = 2.8].

## Discussion

We found that humans recalibrate their perception of visuo-motor simultaneity both to vision-lead and to movement-lead discrepancies, despite the causal asymmetry that the voluntary and intentional finger movement introduces into the scenario investigated (i.e., causes precede possible effects). As a consequence of this asymmetry, the temporal recalibration to movement-lead discrepancies found here and reported by other groups (Stetson et al., 2006; Heron et al., 2009; Sugano et al., 2010) has the counter-intuitive implication that a very fast visual feedback event may, after training, be perceived to precede the movement event that caused it (Stetson et al., 2006; Heron et al., 2009). A similar finding on a perceived temporal reversal of cause and effect has been anecdotally reported in a study on adaptation to visual feedback delays in a motor control task (Cunningham et al., 2001). The experiment presented here, by contrast, also studies the inverse scenario, i.e., adaptation to vision-lead temporal discrepancies. By analogy, a visual event that really occurs shortly before the button press, starts off as with said violation of temporal order of cause and effect and may, after adaptation to vision-lead discrepancies, be interpreted as sensory feedback causally linked to and following up to the intentional action. We found that, despite the causally asymmetrical starting conditions, the mechanisms of temporal recalibration operate in both directions. The results do not give any hint that there is an asymmetry of recalibration around the point of actual visuo-motor simultaneity.

An inherent problem in the study of perceived visuo-motor simultaneity with intentional action is the necessity to present stimuli with vision-lead SOAs. In order to generate comparison stimuli or training stimuli that precede an intentional action, the timing of this action has to be predicted (cf. Stetson et al., 2006; Heron et al., 2009; Sugano et al., 2010; Arnold et al., 2012). Here we present a new method for the presentation of visual stimuli before an intentional action: by recording early movement onset and analyzing it online, the time of a button press can be predicted. As evident from the results, this prediction method is sufficiently accurate to provide visual feedback for recalibration studies (cf. Figure 2). The method presented here does not involve a perceptible lead event such as an external cue (Stetson et al., 2006) or a previous action (Stetson et al., 2006; Heron et al., 2009; Sugano et al., 2010) that could potentially bias a participant’s perceptual judgments. However, even if there is no clearly perceptible lead event in the current experiment, the fact that events happen reliably before a self-initiated action potentially harbors the possibility that participants derive the existence of a non-perceptible lead event that could trigger visual lead stimuli, such as a change in the sensitivity of the button. In the current study, we did not explicitly measure the perceived causal or intentional binding. Therefore, it remains an open question whether the symmetry of recalibration is preceded and catalyzed, accompanied or followed by an analogous change in causal or intentional binding. Further experiments will be necessary to elucidate the link between intentional binding and temporal recalibration of perceived visuo-motor simultaneity.

It should be pointed out that what we and others refer to as visuo-motor temporal recalibration really involves a number of senses. A voluntary movement usually involves at the very least a motor signal (i.e., an efference copy) and proprioceptive feedback. Additionally, given that a button press provides haptic feedback, the tactile sense may play a role in the reported recalibration, given that the visual stimulus is shifted relative to all the mentioned senses. It is unlikely that the recalibration observed in this kind of visuo-motor recalibration paradigm is only due to visuo-tactile or visuo-proprioceptive recalibration. The effect size reported for visuo-motor recalibration (23 ms here; 30–44 ms in Stetson et al., 2006; Heron et al., 2009; Sugano et al., 2010) is much larger than that reported for visuo-tactile only recalibration (12.5 ms; Keetels and Vroomen, 2008) or for visuo-tactile-proprioceptive recalibration (16 ms; Stetson et al., 2006; effect approaching significance). However, it cannot be ruled out that visuo-proprioceptive or visuo-tactile recalibration play a role in the visuo-motor recalibration reported here and it may, therefore, have been more appropriate to use the term “visuo-somatosensory,” referring to the whole complex of non-visual senses involved.

The recalibration we found was preserved between measurement sessions (i.e., across several days). This was an unexpected result. We had assumed that participants would quickly readjust their mapping of visual and motor stimuli after our experiment terminates; interacting with the real world in real time should counter the adaptation experienced in the setup. This unexpected finding suggests that the kind of recalibration observed is specific to the task or device and would likely not transfer to other devices. This is not the only case in which recalibration of perception and action appeared to be highly context-specific and long lasting (e.g., Ernst et al., 2000). High context-specificity may be more common than one would assume in this kind of paradigm.

We also found that good perceptual precision (low JND) appears to decrease the strength of recalibration. This observation is inconsistent with predictions of a Kalman filter model of recalibration. For instance, Burge et al. (2008) found that a decrease of precision of a feedback signal (higher measurement noise) slows down adaptation and could model these effects with a statistically optimal Kalman filter. Such a model would predict the opposite effect that we observe here, i.e., that good precision (low JND) would increase recalibration. It is more likely that participants with low JND recalibrated less because they were able to detect the temporal discrepancy between the timing of the visual stimulus and that of the movement event. The screening for participants with good perceptual precision (JND < 150 ms) means that the participant population tested is more reliable in their perception of time than a random sample of the population, which could thus explain why the amount by which participants’ PSS shifted as a result of recalibration was lower than that reported in previous studies (Stetson et al., 2006; Heron et al., 2009; Sugano et al., 2010).

In conclusion, we found that humans recalibrate their perception of simultaneity of a voluntary action and vision both if the visual event leads and if it lags. A number of factors (session order, perceptual accuracy) appear to modulate the recalibration process. Surprisingly though, there is no evidence that the direction of the temporal discrepancy (vision-lead or vision-lag) is one of them, despite the causal asymmetry that suggests a weaker intentional or causal binding and thus a weaker unity assumption in the vision-lead condition. The mechanisms of temporal recalibration in visuo-motor simultaneity perception (e.g., Stetson et al., 2006; Heron et al., 2009; Sugano et al., 2010; Keetels and Vroomen, 2012; Sugano and Vroomen, 2012) appear to work both forward and backward in time and the data presented here suggests that recalibration may even be symmetrical around the actual point of simultaneity.

## Conflict of Interest Statement

The authors declare that the research was conducted in the absence of any commercial or financial relationships that could be construed as a potential conflict of interest.

## Footnotes

^1^A possible exception is a study by Kato et al. (2009) that presumably found evidence for vision-lead adaptation with very small temporal discrepancies (15 ms). To our knowledge, this research has not been published in article format.
